# Post-Activation Brain Warming: A 1-H MRS Thermometry Study

**DOI:** 10.1371/journal.pone.0127314

**Published:** 2015-05-26

**Authors:** Mario Rango, Cristiana Bonifati, Nereo Bresolin

**Affiliations:** 1 Department of Neurological Sciences, IRCCS Ca’ Granda-Ospedale Maggiore Policlinico Foundation, University of Milan, Milan, Italy; 2 Magnetic Resonance Spectroscopy Center, IRCCS Ca’ Granda-Ospedale Maggiore Policlinico Foundation, University of Milan, Milan, Italy; University Medical Center Goettingen, GERMANY

## Abstract

**Purpose:**

Temperature plays a fundamental role for the proper functioning of the brain. However, there are only fragmentary data on brain temperature (T_br_) and its regulation under different physiological conditions.

**Methods:**

We studied T_br_ in the visual cortex of 20 normal subjects serially with a wide temporal window under different states including rest, activation and recovery by a visual stimulation-Magnetic Resonance Spectroscopy Thermometry combined approach. We also studied T_br_ in a control region, the centrum semiovale, under the same conditions.

**Results:**

Visual cortex mean baseline T_br_ was higher than mean body temperature (37.38 vs 36.60, P<0.001). During activation T_br_ remained unchanged at first and then showed a small decrease (-0.20 C°) around the baseline value. After the end of activation T_br_ increased consistently (+0.60 C°) and then returned to baseline values after some minutes. Centrum semiovale T_br_ remained unchanged through rest, visual stimulation and recovery.

**Conclusion:**

These findings have several implications, among them that neuronal firing itself is not a major source of heat release in the brain and that there is an aftermath of brain activation that lasts minutes before returning to baseline conditions.

## Introduction

Temperature plays a fundamental role for the proper functioning of the brain. Changes in oxygen consumption, changes in cerebral blood flow (CBF), changes in the temperature of incoming arterial blood, and extensive heat exchange between the activated and the surrounding brain tissue [1] are the major factors contributing to brain temperature (T_br_) regulation during functional stimulation when CBF, brain metabolism as well as oxygen extraction increase. Therefore, mechanisms that reduce heat together with others that increase local heat are at work. Under these conditions is difficult to predict the net heat balance resulting in T_br_ because these changes do not occur simultaneously. Based on the Pennes’ bioheat approach [2], temperature distribution in the human brain is predicted to vary inherently within the brain tissue and that activation influences the local temperature according to the anatomical site [3].

Models of brain thermoregulation [2, 3] predict that the surface of the brain would become warmer, whereas the brain temperature in the deeper brain parenchyma may be lower with activation, and that the effects would be greater near the brain surface.

Some experimental findings contradict calculations from mathematical modeling with no clear explanation for this. Brain temperature measurement in animals have shown either an increase [4, 5] or a decrease [6] in temperature during stimulation. Sensory stimulation results in T_br_ changes in either direction, partly depending on the anatomical location. For example, in superficial rodent brain regions (scalp removed but skull intact), infrared optical thermometry techniques showed consistent increases in T_br_ after brain activation [4]. The changes observed were small, 0.18°C, with slow (>10 s) time constants. Other studies in animals, instead, have shown increase of 2–3°C during stimulation [5].

The same infrared approach has demonstrated an increased temperature in the occipital region of the human head during visual stimulation. [7]. These observations indicate that there may be variations, in terms of both polarity and size, in the T_br_ due to brain activation.

Using 1H magnetic resonance spectroscopy (1-H MRS) to estimate noninvasively the human T_br_ inside the occipital lobe, Katz-Brull et al. [8] did not observe significant changes in the short-lasting visual stimulation paradigm. However, they calculated T_br_ from averaged spectra blocks of 16 seconds, that is, with a rather short temporal observational window. Moreover, they kept a short intercycle period that did not allow the return to basal brain conditions between consecutive cycles, therefore adding up the effects of each cycle on brain energetics, blood flow and, eventually, on T_br_. Yablonskiy et al. [9] determined the proton resonance frequency change in the human visual cortex using localized double-spin echo MRS to estimate temperature change. They observed a highly variable change of T_br_ in the visual cortex in response to stimulation, with an average reduction of 0.28°C. However, their MRS method did not utilize a reference resonance frequency that remains stable at different temperatures, such as N-acetylaspartate (NAA), and therefore their results may be affected by the overall magnetic field frequency shifts.

Neither consistent experimental data nor mathematical modeling are available on T_br_ in the post activation/ recovery phase.

In order to overcome the limitations of previous studies and to clarify contradictory results, we planned a study in which T_br_ was observed with a large temporal window, serially, across different functional states, that is, rest, activation and recovery from activation in the visual cortex by a non invasive visual stimulation 1-H MRS combined approach in normal subjects. We used 1-H MRS since it is the most reliable non invasive method for brain thermometry (1).

## Methods

Twenty normal subjects were studied. Their ages were 25–53 years (mean 32 years, SD 9). There were 10 men and 10 women. Normal participants had no history of neurological disease and no history of major disease, vascular risk factors were excluded.

Full ophthalmologic and neurological examinations, standard T1 (Te 10 ms; TR 500 ms), T2 (Te 90 ms; TR 2500 ms), and proton density-weighted (Te 30 ms; TR 2500 ms) MRI brain studies, electroencephalogram, flash, and pattern reversal visual evoked potential were performed to exclude subjects with any of the following: symptoms or signs of central nervous system involvement on neurologic examination, abnormal brain MRI studies, abnormal electroencephalogram, abnormal ophthalmologic examinations, and abnormal visual-evoked potentials. Subjects taking drugs of any kind were excluded.

All participants gave written informed consent. All consent forms were filed within our institution. The project and the consent form have been approved by the national institute of health (Ministero della Salute) and by the local ethical committee (Fondazione Ca’ Granda IRCCS Ospedale Policlinico). The procedures followed were in accordance with the ethical standards of the responsible committee on human experimentation (institutional, Fondazione Ca’ Granda IRCCS Ospedale Policlinico) and with the Helsinki Declaration of 1975 (and as revised in 1983). We did obtain specific consent for the publication of medical images in the figures.

### Visual stimulation

**Fig 1:**
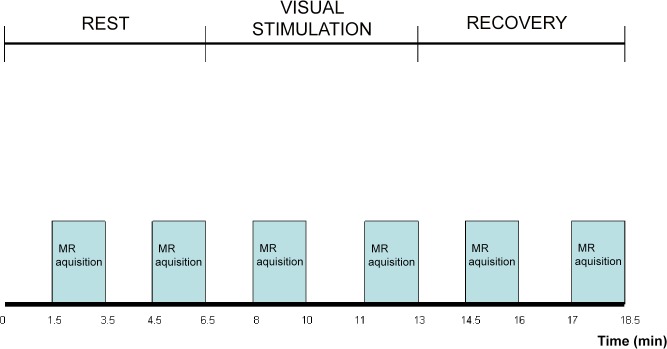
MR brain spectra acquisition for temperature measurement. On the x axis time is represented (minutes). The bars show the time during which each spectrum is acquired, at rest, during visual stimulation and recovery. Each bar corresponds to one spectrum and to one temperature measurement. The entire study takes 18.5 minutes for each subject.

The method used to deliver visual stimulation has been fully described [10]. Briefly, the subject wore goggles (Grass, *www.grasstechnologies.com*) that flashed at 8Hz to reproduce a stimulation pattern used in the previous work [10]. The authors used an infrared-driven computerized system that they developed connected to the MR system computer by which goggles were automatically turned on after 6.5-min acquisition and turned off after 13-min acquisition(Fig 1). Goggles were made of 24 red light emitting LEDs (each goggle) mounted behind a black grid so that the perceived stimulus was a pattern of red rectangles (3 mm x 3 mm) flashing at 8 hertz with each flash lasting 10 ms (square wave) spatially alternating with non flashing black rectangles(3 mm x 3 mm).

A repeat T1 MR imaging study was obtained at the end of spectra collection and compared with images obtained at the beginning of the spectra collection; all studies of that subject were discarded if head positioning had changed.

Evoked potentials with this kind of stimulation were recorded in a separate session outside the magnet in order to confirm neuronal activation in the visual cortex during visual stimulation. All subject showed electrical visual cortex activation during visual stimulation.

### Body temperature measurement

Temperature on the skin of the forehead over the temporal artery and axillary body temperature were measured in all subjects. The temperatures with the two methods correlated significantly (P<0.001) with the mean temporal artery slightly higher than mean axillary temperature (36.9 vs 3.6, p<0.01).

Temperature on the skin of the forehead over the temporal artery was measured by an infrared electronic thermometer (Exergen Temporal Scanner). Axillary body temperature was measured by a mercury-in-glass thermometer. Temperature was measured in patients and in control participants laid on the MRI bed (outside the magnet) immediately before and after the MR study.

No febrile participant at the time of the study (body temperature >37°C with any of the three methods) was included in the study. Before the MRS study, participants sat in the waiting hall of the MRI unit for 60 min before their MRS assessment to ensure proper adaptation. The hall’s temperature was kept constant at 22°C throughout all the assessment periods. The magnet room temperature was kept at 22°C.

All the studies were performed on the same day of the week between 2 and 4 p.m.

### Brain temperature measurement

All studies were performed on a 1.5T Siemens System (Siemens, Erlangen, Germany) using a quadrature detection transmitter/receiver (Tx/Rx) head coil, and the images were assessed under blinded conditions by an MRI expert in order to exclude abnormal MRI.

MR sagittal, axial, and coronal T1 and T2/proton density weighted images were acquired. Single-voxel 1H-MRS spectra were acquired without water suppression from a volume of interest (20x20x10mm) aligned along the calcarine sulcus of the occipital cortex and in the right centrum semiovale, in which no visual fibers transit. A point-resolved spectroscopy sequence (PRESS) was used. The acquisition parameters were TR 2000 ms, TE 270 ms, 64 acquisitions, 1024 points per spectrum, 1000 Hz spectral width.

Spectra were acquired at rest (baseline condition), during visual stimulation and after the end of visual stimulation (Fig 1).

Two spectra from the visual cortex were acquired: between 1.5 and 3.5 minutes and between 4.5 and 6.5 minutes after the beginning of each state (Fig 1). Similarly two spectra were then acquired from the centrum semiovale between the 1.5 and 3.5 minutes and between 4.5 and 6.5 minutes after the beginning of each state: since the mean group values of the two spectra at rest did not differ statistically both for the visual cortex (p>0.1) and for the centrum semiovale (p>0.1), the two temperature values obtained from the rest state were averaged and one single value obtained for each subject and reported as such.

JMRUI software (URL http://www.mrui.uab.es/mrui/) was used for spectra analysis. All spectra were inspected visually first and discarded if judged to be of poor quality (e.g. having a badly elevated baseline or containing spurious peaks). Spectra were also discarded if line widths at half maximum were greater than 8 Hz or if the metabolite peaks were more than 0.1ppm offset from their expected values. Data processing included Lorentzian filtering (1 Hz line broadening), Fourier transformation, and automatic phase correction.

**Fig 2:**
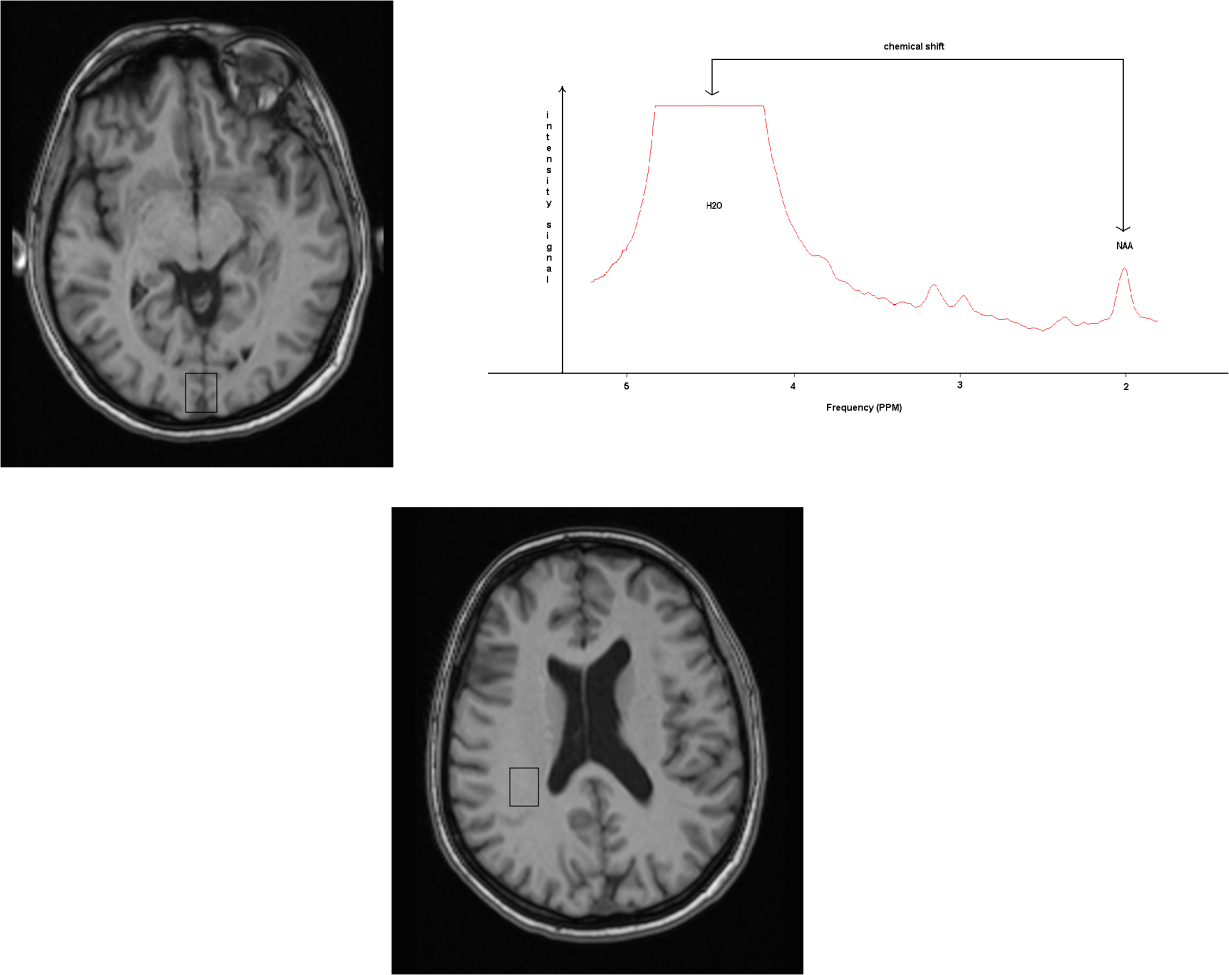
Visual cortex spectrum and brain temperature measurement. Voxel of 20x20x10 mm centred on the calcarine sulcus of a subject (A) from which a 1H MR unfitted spectrum (B) is obtained. Spectra acquisition parameters: PRESS sequence, TR 2000 ms, TE 270 ms. The MR signals corresponding to water and N-Acetyl-Aspartate are identified (respectively H2O and NAA). The chemical shift used for temperature calculations is shown by arrows. (C) shows the centrum semiovale voxel of 20x20x10 mm.

A typical spectrum obtained from the visual cortex is displayed in Fig 2.

The temperature calculation was based on the measurement of the difference between the water resonance frequency, which is linearly temperature dependent, and the NAA methyl resonance frequency, which remains stable at different temperatures [11, 12, 13].

Absolute values of T_br_ (expressed in Celsius degrees) were calculated according to the equation T = 228.2–72.2 x (δ_H2O_ – δ_NAA_) [12], where δ_H2O_ and δ_NAA_ are the resonance frequencies expressed in ppm, measured at the center of the water and of the NAA fitted peaks. Absolute values of T_br_ (expressed in Celsius degrees) were also calculated using Cr as internal reference according to the equation T = 36+a(fw-fref-f0)°C, with a = -101.7°C/ppm, f0 = -1.6585 ppm for Cr where fw and fref are the water and Cr chemical shifts (ppm), respectively, and f0 is the chemical shift difference between water and the reference at 36°C [14].

We utilized a time domain analysis using jMRUI software in two steps. First, the water signal was modeled as a mono-exponentially decaying sinusoid (a single component) using the Hankel Lanczos Total Least Squares (HLTLS) algorithm (14) and its resonance frequency estimated.

Second, the water signal was removed (filtered) from the FID by Hankel Lanczos Singular Value Decomposition (HLSVD) algorithm [14] in a 2-ppm range around the water resonance frequency. The residual signal was then used to estimate the NAA resonance frequency using the HLTLS algorithm.

The sensitivity of this MR thermometry method is 0.0103 ppm/1°C. The actual sensitivity and accuracy were determined in a phantom experiment (Table 1). Fiber optic temperature sensors were calibrated at room temperature, relative to a type T copper/constantan thermocouple and thermometer.

**Table 1:** Comparison between fiber optic thermometry and MRS thermometry in a brain-like phantom.

Fiber optic	**36.80**	**36.85**	**36.90**	**36.95**	**37.00**	**37.05**	**37.10**	**37.15**	**37.20**	**37.25**
MRS	36.80	36.80	36.90	37.00	37.00	37.00	37.10	37.10	37.20	37.30

The first row reports fiber optic measured phantom temperatures, the second row reports MRS measured phantom temperatures. Temperatures are reported in °C.

Sensors were inserted within a spherical phantom simulating the human brain size and shape which contained a water solution of NAA, Cr, Cho, My, Glx, albumin at concentrations as in the human brain.

Phantom was heated at 38°C and kept at this temperature by a thermal blanket with thermostatically controlled circulating water passing through it.

Appropriate coefficients and constants were obtained and inserted within the equation for MRS temperature calculations in order to calibrate the MRS system so that MRS thermometry measured also 38°C.

Afterwards, the phantom was allowed to cool down at a speed of 0.1°C/ four minutes, and for every 0.1°C step MRS thermometry measurement was repeated (2 minutes measurement). The formula and the methods for MRS thermometry were as reported in the methods. The table reports the measurements between 36.80 and 37.25°C showing that there is agreement between fiber optic and MRS thermometry with an accuracy and sensitivity within 0.1°C. The same kind of agreement was present in all the measured interval, that is, 36.40–38.20°C. Since the linewidth in vivo is larger, we corrected the phantom sensitivity by the ratio of the linewidth in vivo/in vitro and calculated a final actual temperature sensitivity as well as accuracy in vivo within 0.2°C.

For these reasons we rounded brain temperature values at 0.20°C steps.

Two-tailed *t*-tests, analysis of variance (one-way ANOVA,; degrees of freedom 4, 76) and r correlation coefficients (Pearson’s r) were used for statistical analysis (NCSS software). Bonferroni’ s correction for multiple comparisons was applied.

## Results

### Body temperature

The mean axillary body temperature did not differ between the beginning of the study and the end of the study (mean: 36.6°C, 0.12 SD vs 36.5°C, 0.11 SD, P = >0.1). Likely, the temperature on the skin of the forehead over the temporal artery did not differ between the beginning of the study and the end of the study (mean: 36.9°C, 0.11 SD vs 36.9°C, 0.12 SD, P = >0.1).

### Visual cortex temperature

Single values across the different conditions are reported in Table 2, mean values are reported in Table 3.

**Table 2:** Visual cortex brain temperature single values.

Subjects	Rest	Visual	Visual	Recovery 1	Recovery 2
		stimulation 1	stimulation 2		
**1**	37.40	37.40	37.20	38.00	37.60
**2**	37.20	37.20	37.20	37.60	37.20
**3**	37.40	37.40	37.40	37.60	37.40
**4**	37.80	37.80	37.40	38.00	38.00
**5**	37.20	37.20	37.00	37.60	37.00
**6**	38.00	37.80	37.60	38.00	38.00
**7**	37.40	37.40	37.20	37.20	37.20
**8**	37.60	37.40	37.20	37.80	37.80
**9**	37.60	37.80	37.40	38.00	37.60
**10**	37.40	37.40	37.40	37.80	37.60
**11**	37.00	37.00	36.80	37.40	36.80
**12**	37.80	37.60	37.40	38.20	37.80
**13**	37.20	37.40	37.40	37.60	37.40
**14**	37.40	37.80	37.20	37.80	37.40
**15**	37.80	37.80	37.60	38.20	37.60
**16**	37.20	37.40	37.20	37.20	37.20
**17**	37.40	37.40	37.40	37.60	37.40
**18**	37.00	37.00	36.80	37.60	37.20
**19**	37.80	37.60	37.20	38.20	37.60
**20**	37.40	37.40	37.60	37.40	37.40

Each of the 20 rows corresponds to a different subject: for each patient brain temperature in the visual cortex is reported through the five different states (rest, first part of visual stimulation, second part of visual stimulation, first part of recovery, second part of recovery).

**Table 3:** Brain temperature mean values

	Rest	Visual stimulation		Recovery	
		First part	Second part	First part	Second part
**Visual cortex**	37.40 (0.20)	37.40 (0.20)	37.20 (0.20)	37.80 (0.20)	37.40 (0.20)
**Centrum semiovale**	37.60 (0.20)	37.60 (0.20)	37.60 (0.20)	37.60 (0.20)	37.60 (0.20)

Values are reported as °C (group mean), in parentheses SD.

Values are rounded at 0.20 °C steps.

T_br_ at rest was higher than body temperature (temporal artery) (mean: 37.40°C vs 36.90°C, P<0.001).

An ANOVA analysis revealed a significant difference of the mean temperature across the five states (rest, visual stimulation 1, visual stimulation 2, recovery 1, recovery 2) (P<0.001).

### Rest

No difference was found between the means of the first and the second measurement at rest since the values from the two measurements were overlapping (P>0.1).

Mean T_br_ was:

(Water-NAA shift based) 37.40°C (SD 0.20), (Water-Cr shift based) 37.40°C (SD 0.20), t-test P>0.1.

### Visual stimulation

At 2 minutes after the beginning of visual stimulation mean T_br_ remained unchanged: (Water-NAA shift based) 37.40°C (SD 0.20) (Table 3), (Water-Cr shift based) 37. 40 (SD 0.20), t test P>0.1. At 4 minutes mean T_br_ decreased to: (Water-NAA shift based) 37.20°C (SD 0.20) (0.20°C below the baseline value, P<0.05) (Table 3), (Water-Cr shift based) 37.20 (SD 0.20), t test P>0.1.

### Recovery

Mean T_br_ at 2 minutes after the end of visual stimulation rose significantly to: (Water-NAA shift based) 37.80°C (SD 0.40) (0.40°C above baseline value, P<0.0001 and 0.60°C above the value reached during the second part of visual stimulation) (Table 1), (Water-Cr shift based) 37.80 (SD 0.40). At 4 minutes after the end of visual stimulation mean T_br_ was back to (Water-NAA shift based) 37.40 (SD 0.20) thus overlapping the baseline value (P>0.1)(Table 3), (Water-Cr shift based) 37.40 (SD 0.20) t test P>0.1.

### Centrum semiovale temperature

No difference was found between the first and the second measurement at rest since the values from the two measurements were overlapping (P>0.1).

An ANOVA analysis did not reveal difference of the mean temperature across the five states (rest, activation1, activation 2, recovery 1, recovery 2) (P>0.1).

At rest mean T_br_ was (Water-NAA shift based) 37.60°C (SD 0.20), (Water-Cr shift based) 37.60 (SD 0.20) (Table 2), t test: P>0.1.

Mean T_br_ at 2 minutes after beginning of visual stimulation was 37. 60 (SD 0.20)(P>0.1) and at 4 minutes mean T_br_ was 37. 60 (SD 0.25) (P>0.1).

Mean T_br_ at 2 minutes after the end of visual stimulation was 37.60 (SD 0.20)(P>0.1) and at 4 minutes after the end of visual stimulation mean T_br_ was 37. 60 (SD 0.20)(P>0.1).

## Discussion

This is the first study observing sequential brain temperature variations across different functional states. We used a large temporal window since the time constant of the temperature change is of several seconds to minutes [5].

T_br_ at rest was higher than body temperature in line with previous measurement [1]. The T_br_ of the stimulated region at first remained stable and then decreased slightly. These findings help to explain contradictory results of short stimulation studies [15] versus longer stimulation studies [16]. We chose to study the visual cortex because visual activation of the brain appears optimal given the high oxidative metabolism [4, 11] and high levels of oxidative phosphorylation enzymes in the visual cortex [17]. Additionally, visual stimuli can be easily graded [18].

Intense heat production is an essential feature of brain energetics, with most of the energy used for brain functioning eventually released as heat [19]. In the brain, heat is produced mostly by mitochondrial oxidative chemical reactions. Basal heat production within the brain is high and is dissipated through the circulation and by conduction through the skull. Most of the heat is dissipated through CBF, with venous blood leaving the brain at a higher temperature than the incoming arterial blood [1, 19].

Under normal circumstances at interior locations in brain, increases of CBF are expected to reduce T_br_ whereas increases in metabolism are expected to increase T_br_ (1).

Glycolisis, that releases less heat per mole of glucose than oxidative respiration, seems to prevail in the first part of brain activation whereas respiration prevails in the second part [20]. During functional activation, CBF increases significantly, and brain metabolism also increases but it is unclear the exact timing of these changes and their relationship.

During activation, our data may be explained by a change of the ratio between CBF and glucose or oxygen metabolism [19–22]. Previous work has shown that CBF returns to the baseline almost immediately after the end of brain activation [21], whereas oxygen metabolism as well as glucose metabolism remain elevated longer [21, 22]. As a result, an excess of heat is released from the brain that is not adequately removed by increased CBF. This explains why T_br_ increases in the first 3–4 minutes of the post activation phase and then returns towards baseline values afterward when oxygen metabolism decreases [21]. This is the first report showing that in humans T_br_ increases once activation is over, that is, after the end of increased neuronal firing. This finding has several implications. First, neuronal firing is not a major source of heat release itself. Second, it shows that there is an aftermath of the increased brain activity that extends for several minutes beyond the activation phase itself and that this aftermath is dynamic with a progressive return to baseline conditions. This must be taken into account if a new stimulation is delivered to the brain before a proper post activation/ recovery interval is observed: in this case the starting conditions differ from when a proper post activation interval is observed. This may be important in the planning of MR BOLD studies since oxygen release from hemoglobin is temperature dependent [15, 16].

Temperature influences several biochemical and physiological parameters involved in brain temperature control [1]. The change of 0.60°C that we found in the visual cortex temperature between the second part of visual stimulation and the first part of recovery represents physiologically a very consistent temperature change in a short time, that may heavily influence brain functioning. In studies on perfused brain slices, temperature increases enhance oxygen metabolism [1]. The consequences of changes in brain temperature on CBF are somewhat controversial, with some evidence that selective brain cooling increases CBF above control levels [1].

Most physical and chemical processes are affected by temperature, with various effects of temperature changes on passive membrane properties, single neuronal spike, and spike bursts, and on the neuronal responses induced by direct and indirect electric stimulation of tissue or its afferent pathways [1, 23–25].

Small temperature variations affect protein conformation and assembly, and alters protein expression [1, 23]. Temperature also strongly modulates neurotransmitter release [1, 25].

Neuronal activity is temperature dependent with neuronal firing increasing with increased temperature [1, 24]. As a consequence, brain may be more ready to activate immediately after activation than at rest. The increase of glucose and oxygen metabolism in the post activation phase is explained, at least partly, by increased local temperature. In fact, chemical reactions are temperature dependent with high temperature increasing brain metabolism [1, 26].

The maintenance of a stable temperature is of utmost importance for the proper functioning of the brain. The brain is the most heat-sensitive organ in the human body [1], and mitochondrial and plasma membranes are the most temperature-sensitive cellular elements with hyperthermia potentiating the cytotoxic effects of reactive oxygen species [1, 23]. Some diseases are worsened by increased temperature. For example, multiple sclerosis symptoms worsen when environment temperature increases. Seizure also are induced by increased body temperature as well as by visual stimulation. It cannot be ruled out, therefore, that repeated prolonged brain stimulation are deleterious in these diseases and studies to investigate this should be performed.

Some technical issues on MR thermometry worth discussing. It has been reported that frequency differences for water on the order of 0.014 ppm may be found between WM, GM and CSF [27]. For this reason we used the same method as in the the original papers by Cady and Corbett where they used temperature sensors and validated the MRS thermometry in a tissue that contained a mixture of gray and white matters, as we did in our study investigating a mixture of gray and white matter around the visual cortex. We also studied white matter alone: however the purpose here was to detect whether temperature would change in the different states independently from the absolute temperature value and not to determine its absolute value. Moreover, we obtained temperatures from the shift between water and Cr, that, unlike the water-NAA distance, is not dependent [27] on the tissue studied and we found overlapping results. Also, it was reported in [28] that NAA peak actually consists of two peaks—NAA and NAAG shifted by about 0.03ppm. However, NAAG concentration in normal brain tissue is somewhere between one third-fifth to 1 tenth of NAA [28, 29], therefore is not likely to influence significantly NAA peak frequency and therefore temperature measurement. Pragmatically, temperature measured from water Cr shift did not differ from temperature measured by water NAA shift in our study. Blood magnetic susceptibility changes during functional activation (BOLD effect). can also cause, in theory, a change in the resonance frequencies of water and NAA. However, a paper from Zhu and Chen [30] shows that bold effect affects equally water and NAA in terms of linewidth and height this meaning the susceptibility changes induced by BOLD affect in the same manner water and NAA. Therefore, we expect also frequency to be equally affected for water and NAA with the frequency distance between water and NAA being unaffected as a result.Moreover, the BOLD effect affects, concomitantly, and, almost immediately, gray and white matter in the same direction within a single state, meanwhile temperature changes only after some minutes of activation, this suggesting that frequency changes are actually, mostly, temperature dependent.

In summary, visual cortex T_br_ during activation T_br_ remains unchanged at first and then shows a small decrease around the baseline value. After the end of activation T_br_ increases consistently and then returns to baseline values after some minutes. Centrum semiovale T_br_ instead remains unchanged through rest, visual stimulation and recovery.

It follows that neuronal firing itself is not a major source of heat release in the brain and that there is an aftermath of increased brain firing that lasts minutes before returning to baseline conditions.
